# A Case of Recurrent Compartment Syndrome with Concomitant Use of Eliquis

**DOI:** 10.1155/2022/1863538

**Published:** 2022-03-07

**Authors:** James A. Nemunaitis, Jason P. Den Haese, Mark S. Buseck, Shawn W. Storm, Joshua A. Tuck, Anthony J. Ferretti

**Affiliations:** ^1^Department of Orthopedic Surgery, LECOM Health Millcreek Community Hospital, 5515 Peach St, Erie, PA 16509, USA; ^2^Lake Erie College of Osteopathic Medicine, 1858 W Grandview Blvd, Erie, PA 16509, USA; ^3^Saint Vincent Orthopedic Institute, 2315 Myrtle St, Erie, PA 16502, USA

## Abstract

*Introduction*. Acute compartment syndrome (ACS) occurs secondary to increasing pressure within a fascial compartment that exceeds perfusion pressure. This can be caused by spontaneous hematomas, which can be secondary to prolonged anticoagulation therapy. Eliquis® has not been associated with ACS of the thigh in any of the currently published literature. Identifying ACS early is important because it can reduce the risk of permanent structural damage, limb amputations, and mortality rates. *Case Report*. A 43-year-old male with past medical history of unprovoked Deep Vein Thrombosis (DVT) eight months prior to presentation on Eliquis® presented to the emergency department for significant right thigh pain after riding a roller coaster. There was increased tone/firmness of the anterior compartment and tenderness on palpation of the proximal two-thirds of the anterior thigh. Imaging, clinical findings, and Stryker needle measurements confirmed ACS secondary to hematoma, which required fasciotomy and evacuation of the hematoma. The patient was temporarily switched to aspirin for DVT prophylaxis postoperatively to prevent new hematoma formation. Six weeks later, the patient arrived at the ED with a DVT that was treated with Eliquis®. Eight months later, the same patient presented with acute right thigh pain that started while lying in bed. A diagnosis of recurrent ACS in the right anterior thigh was made, requiring a fasciotomy. Surgery was successful without any complications. *Discussion*. Eliquis® is associated with an increased risk of hematoma formation, which can lead to ACS. This is a rare adverse effect that providers should be aware of because it requires early management to prevent ACS-associated complications. This is significant because no currently published literature has identified an association of Eliquis® with ACS in the thigh. In cases of atraumatic ACS, we were unable to find any protocols advocating for or against the use of DVT prophylaxis postfasciotomy in the literature.

## 1. Introduction

Acute compartment syndrome (ACS) is a surgical emergency that occurs when there is an increase in pressure within a fascial compartment that exceeds perfusion pressure. The resulting localized ischemia leads to cellular anoxia and necrosis of muscular and neurovascular structures within the compartment [1]. It is a clinical diagnosis with five or six major signs described and referred to as the five P's or six P's of compartment syndrome: pain out of proportion, pain with passive stretch, paresthesia, paralysis, pulselessness, and poikilothermia.

Pain out of proportion and pain with passive stretch are the most common early findings of ACS, with the latter being the most sensitive clinical finding [2]. Paralysis, pallor, and absent pulses are late findings that often present after permanent damage has occurred. These symptoms are not all always present, and none may be present in an obtunded or sedated patient. The diagnosis of ACS may be confirmed by measuring the compartments with a manometer or Stryker needle if it does not delay fasciotomy. Measuring compartment pressures is especially critical in patients that are obtunded or sedated and therefore cannot participate in the exam. Absolute compartment pressure ≥ 30 mmHg or delta pressure (diastolic BP prior to anesthesia minus compartment pressure) ≤ 30 mmHg requires surgical intervention with a fasciotomy to preserve limb function and prevent complications [3].

The most common cause of ACS is secondary to long bone (primarily tibial) fractures in the lower extremities, but it may occur in any fascial compartment [1]. A systematic review performed in 2010 revealed that 90% of ACS cases in the thigh involved blunt trauma [4]. Spontaneous hematoma associated with bleeding disorders and anticoagulation has also been associated with traumatic and atraumatic lower extremity ACS [5]. Low molecular weight heparin (LMWH) such as Lovenox® (enoxaparin) [6–9], Marcumar® (phenprocoumon) [10], and Coumadin® (warfarin) [11] has been documented associations with hematomas leading to atraumatic ACS in the thigh. However, published cases of ACS in the thigh with concomitant Eliquis® (apixaban) use have not been cited in current literature. It is important to be cognizant of this correlation because identifying ACS early can reduce the risk of permanent structural damage, limb amputations, and mortality rates [12]. Of these complications, persistent neurologic deficits are the most common [4]. If ACS in the lower extremity progresses to require a fasciotomy, mortality rates range from 11 to 25% [3].

## 2. Case Presentation

A 43-year-old male with past medical history of unprovoked Deep Vein Thrombosis (DVT) eight months ago on Eliquis® 5 mg twice daily presented to the emergency department (ED) for right thigh pain after riding a roller coaster three hours prior. He reported pain in the thigh from the lap bar during the ride. He initially could walk, but then the pain became so bad that he had to use a wheelchair to get back to his car for a family member to drive him to the hospital. There was significant increasing anterior and lateral thigh pain for which he was initially given 4 mg morphine, with minimal improvement. A subsequent dose of 0.5 mg Dilaudid® (hydromorphone) did not relieve the pain, which had become much worse at the time. There was no posterior or medial thigh pain present. The patient had a previous injury to the same thigh as a child. He was observed overnight for compartment syndrome but did not require surgery. The patient had been taking Eliquis® 5 mg twice daily for eight months, following his first unprovoked DVT. He had no family history of DVT or other known risk factors.

On physical examination, the patient appeared moderately distressed secondary to right lower extremity pain. There was generalized swelling of the anterior and lateral thigh without ecchymosis. There was increased tone/firmness to the anterior compartment with tenderness. The posterior and medial thighs were soft and compressible. Passive flexion of the knee past 20 degrees was limited by pain. The extensor mechanism was intact with 10 degrees of extensor lag. There was no pain on abduction of the hip. Strength testing of knee flexion and extension was 3/5 and limited secondary to pain. Ankle dorsiflexion/plantarflexion and extensor hallucis longus strength were 5/5. Pulses were palpable and symmetric in the bilateral lower extremities. Sensation was intact and equal bilaterally.

A computed tomography (CT) scan of the thigh revealed anterior soft tissue swelling with an intramuscular hematoma predominantly in the vastus medialis muscle, consistent with physical exam findings. Laboratory results were unremarkable. A Stryker needle (Portage, MI 49002, USA) measured an anterior compartment pressure of 42 mmHg.

The clinical presentation of pain out of proportion, CT findings, and elevated absolute compartment pressure confirmed the diagnosis of ACS in the right anterior thigh compartment. A plan was made for treatment with Factor Eight Inhibitor Bypassing Activity nanofiltration (Feiba®) to reverse the effects of Eliquis® in order to prepare for an emergent fasciotomy. Anterior thigh compartment fasciotomy with vacuum assistive closure was successful without any complications. Plastic surgery performed primary wound closure three weeks postoperatively. Aspirin 325 mg daily was chosen for DVT prophylaxis after discussion with vascular surgery to prevent recurrent hematoma formation.

Six weeks postoperatively, the patient presented to the ED with a well-healed surgical incision and painless right lower extremity swelling. A DVT was discovered on venous duplex ultrasound, and the patient was restarted on Eliquis® 10 mg tablet twice daily for seven days followed by 5 mg tablet twice daily for six months. He was then transitioned to a prophylaxis dose of 2.5 mg twice daily after six months of treatment for DVT.

**Figure 1:**
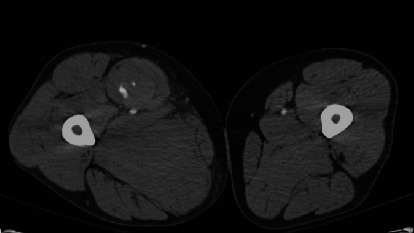
An axial CTA image of bilateral thighs demonstrating a hematoma in the sartorius of the right thigh.

**Figure 2:**
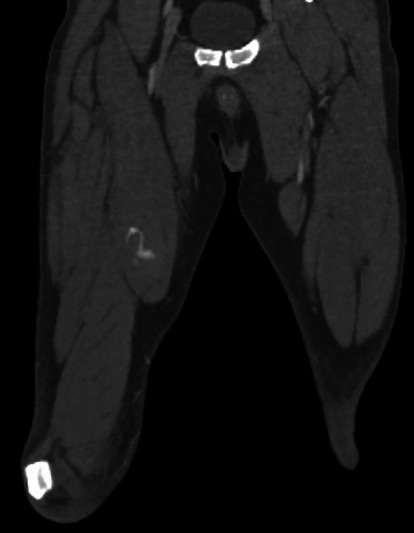
A coronal CTA image of bilateral thighs demonstrating a hematoma in the sartorius of the right thigh.

Eight months postoperatively, the patient presented to the ED with acute right thigh pain that started while lying in bed. On physical exam, there was increased tension felt in the anterior and medial compartments. Computed tomography with angiography (CTA) of the right thigh identified a hematoma with a small active bleed within the sartorius musculature with swelling (Figures 1 and 2).

On exam, the patient was moderately distressed secondary to right lower extremity pain. Swelling of the thigh was present with increased tone in the anterior medial and posterior compartments. Pain was present with global palpation of the thigh compartments. There was also significant thigh pain with passive knee flexion/extension and hip abduction. Distally, strength was 5/5 with ankle dorsiflexion/plantarflexion and great toe extension. Sensation was intact and equal bilaterally. Pulses were palpable and symmetric in the bilateral lower extremities.

Clinical findings were consistent with ACS, and Stryker needle measurements were taken. The Stryker needle measured 31 mmHg in the anterior compartment, 44 mmHg in the medial compartment, and 35 mmHg in the posterior compartment.

The clinical presentation of pain out of proportion, CTA findings, and elevated absolute compartment pressure confirmed the diagnosis of recurrent ACS and involvement of all three compartments. The patient underwent a 2-incision, 3-compartment fasciotomy of the right thigh, and an evacuation of intramuscular hematoma. Primary skin closure was performed. A repeat venous duplex ultrasound of the lower extremities was negative, so Aspirin 81 mg twice daily was chosen for DVT prophylaxis due to the risk of recurrent hematoma. Seven weeks postoperatively, the patient was recovering well with mild paresthesia present over his right knee.

## 3. Discussion

ACS is an orthopedic emergency that can occur in any fascial compartment within the body. Cases of ACS within the lower extremity that require fasciotomies are associated with a mortality rate as high as 25-47% [3, 13]. Therefore, it is important to identify all risk factors that can be associated with ACS; early diagnosis and treatment are vital in preventing unfavorable outcomes such as permanent muscular and neurovascular damage, limb amputation, and death [12]. Anticoagulants used for DVT prophylaxis and atrial fibrillation, such as Lovenox® (enoxaparin) [6–9] and Coumadin® (warfarin) [11], have had published case reports associated with hematomas that lead to ACS in the thigh. To our knowledge, no case reports have been published about compartment syndrome of the thigh while on Eliquis® or recurrent compartment syndrome of the thigh on Eliquis®.

Eliquis® has been associated with a lower risk of bleeding than warfarin [2]. The duration of prophylaxis is also important to avoid overtreatment and an increased risk of postoperative complications. As a physician, this bleeding risk needs to be considered when selecting a drug for anticoagulation therapy. A retrospective study comparing apixaban, warfarin, rivaroxaban, and dabigatran (a direct thrombin inhibitor) showed that warfarin has the highest bleeding risk. Apixaban was found to have a significantly lower GI bleeding risk and no significant difference in overall major bleeding risk than dabigatran [14]. For DVT prophylaxis after orthopedic surgery, apixaban and rivaroxaban had a higher therapeutic index and efficacy than enoxaparin. Of these direct factor Xa inhibitors, apixaban was the only one to show a significant decrease in bleeding risk when compared to LMWH at higher doses [15]. Furthermore, research for other orthopedic surgeries (such as total hip arthroplasty and total knee arthroplasty) showed no significant difference in postoperative DVT when comparing the use of aspirin to other anticoagulants [16, 17].

On review of literature, we were unable to identify any protocols or recommendations clearly advocating for the use of DVT prophylaxis after a fasciotomy in cases of atraumatic ACS. UpToDate states, “The risk of anticoagulation after fasciotomy may outweigh the theoretical benefits for many patients, and there are no convincing data to support its routine use in this setting. Anticoagulation is warranted for acute ischemia and compartment syndrome due to thromboembolism [18]”.

This patient did not receive Eliquis® postoperatively due to its potential risk of causing a recurrent hematoma. Instead, this patient received one month of DVT prophylaxis with aspirin. Two weeks after discontinuation of anticoagulation prophylaxis, the DVT occurred. We believe that the DVT would have occurred regardless of the type of anticoagulant prescribed because it occurred outside the typical window of time that we provide prophylactic therapy. Then, the use of Eliquis® to treat the DVT resulted in a spontaneous hematoma formation and recurrent ACS. Due to this assumption and a negative venous duplex ultrasound, Eliquis® DVT prophylaxis after the second fasciotomy was avoided because there was a high risk of it causing a third hematoma in this patient. This case highlights the need to weigh the risks and benefits of anticoagulant use.

## 4. Conclusion

This case illustrates a serious, though rare, adverse effect that can occur with the use of Eliquis® or other anticoagulants. Spontaneous hematoma formation within a fascial compartment while on anticoagulants may result in increased compartment pressures and ACS. Both instances of ACS in this patient were treated successfully with a fasciotomy and had good outcomes. To our knowledge, there are no published case studies of ACS of the thigh with use of Eliquis®. Early diagnosis and treatment of ACS are imperative for better patient outcomes [12]. In cases of atraumatic ACS, we were unable to find any protocols advocating for or arguing against the use of DVT prophylaxis postfasciotomy. In conclusion, in the setting of anticoagulation use, physicians should include compartment syndrome in their differential diagnosis, even in the case of atraumatic pain within a fascial compartment. Further research is needed to determine the incidence of DVT and recommendations for DVT prophylaxis after fasciotomy.

## Data Availability

Access to EMR data is restricted.
